# Planetary Health Diet, Obesity and Sleep Quality in Italian Adults

**DOI:** 10.3390/nu18213586

**Published:** 2026-10-29

**Authors:** Justyna Godos, Giuseppe Caruso, Alice Rosi, Maria Antonieta Touriz Bonifaz, Francesca Scazzina, Maria Paula Roca-Solano, Florencia Ceriani, Massimiliano Tucci, Daniela Martini, Giuseppe Grosso

**Affiliations:** 1 Department of Biomedical and Biotechnological Sciences, University of Catania, 95123 Catania, Italy; 2 Departmental Faculty of Medicine, UniCamillus-Saint Camillus International University of Health Sciences, 00131 Rome, Italy; 3 IRCCS San Camillo Hospital, 30126 Venice, Italy; 4 Human Nutrition Unit, Department of Food and Drug, University of Parma, 43125 Parma, Italy; 5 Facultad de Ciencias de la Salud, Universidad Católica de Santiago de Guayaquil, Av. Pdte. Carlos Julio Arosemena Tola, Guayaquil 090615, Ecuador; 6 Facultad de Ciencias Médicas, Universidad de Guayaquil, Avenida 10 NO, Guayaquil 090613, Ecuador; 7 Escuela de Humanidades, Universidad Espíritu Santo, Samborondon 0901952, Ecuador; 8 Nutrition School, Universidad de la Republica (UdelaR), Montevideo 11200, Uruguay; 9 Division of Human Nutrition, Department of Food, Environmental and Nutritional Sciences (DeFENS), Università degli Studi di Milano, 20133 Milan, Italy; massimiliano.tucci@unimi.it (M.T.);

**Keywords:** planetary health diet, sustainability, weight status, obesity, sleep

## Abstract

**Background/Objectives:** Sleep and diet have been demonstrated to be interconnected factors playing an important role in human health. However, the relationship between sustainable dietary patterns, sleep, and obesity remains to be fully elucidated. This study investigated the association between adherence to the Planetary Health Diet (PHD) and sleep quality among Italian adults, while also exploring the potential role of weight status in this relationship. **Methods:** This observational study included 1936 Italian adults. Dietary intake was assessed using a validated food-frequency questionnaire (FFQ), and adherence to the PHD was evaluated using a validated index. Sleep quality was assessed using the Pittsburgh Sleep Quality Index (PSQI). Multivariable logistic regression analyses were conducted to examine the associations between PHD adherence and sleep quality. **Results:** Among 1936 participants, higher PHDI adherence was associated with greater odds of adequate sleep quality. Compared with the lowest quartile, participants in the highest PHDI quartile had 65% higher odds of adequate sleep quality (OR: 1.65; 95% CI: 1.20, 2.28); significant associations were also observed for sleep disturbance and self-rated sleep quality, whereas sleep duration, sleep efficiency, and sleep-medication use were not associated with PHDI adherence. Associations were more pronounced among participants with overweight or obesity, in whom the highest quartile was associated with more than twice the odds of adequate sleep quality (OR: 2.40; 95% CI: 1.46, 3.95). Additional domain-specific associations were observed in this subgroup, whereas no significant association with overall sleep quality was found among normal-weight participants. **Conclusions:** Greater adherence to the PHD was associated with better overall and self-rated sleep quality among Italian adults, with stronger associations observed among individuals with overweight or obesity. These findings suggest that adherence to a healthy and sustainable dietary pattern may be related to better sleep health, particularly among individuals with excess body weight.

## 1. Introduction

Sleep is a multidimensional health behavior encompassing duration, continuity, timing, regularity, efficiency, and subjective satisfaction, with sleep quality capturing aspects that are not adequately represented by sleep duration alone [1]. Adequate sleep is demonstrated to contribute to neuroendocrine and metabolic regulation, immune competence, and inflammatory homeostasis [2]. Existing comprehensive evidence links unfavorable sleep characteristics (including short or long sleep duration, insomnia, and sleep-disordered breathing) with obesity, type-2 diabetes, hypertension, and coronary heart disease [3], as well as stroke and dementia [4,5], although the strength and potential causality of these associations varied across sleep traits and outcomes. The cardio-metabolic relation has been reported comprehensively [6,7], whereas prospective evidence indicates that insomnia is associated with the subsequent development of depression [8]. Because much of this evidence is observational, poor sleep may act as both a contributor to and a consequence of deteriorating physical or mental health, and reverse causation remains an important consideration. Body weight is particularly relevant to the relation between sleep and health due to such bidirectional connection [9]. Prospective studies indicate that short sleep duration is associated with a higher incidence of obesity in adults [2]. Experimental and epidemiologic evidence suggests that insufficient or disrupted sleep may affect glucose metabolism, insulin sensitivity, appetite regulation, reward-driven food choice, and energy balance, thereby favoring weight gain and metabolic dysfunction [10,11]. Conversely, excess adiposity can impair sleep through altered respiratory mechanics, upper-airway narrowing, sleep-disordered breathing, pain, and obesity-related metabolic and inflammatory disturbances [12,13]. Body mass index (BMI) may therefore confound, mediate, or modify associations between dietary exposures and sleep outcomes, making analyses across weight-status categories particularly informative.

Sleep is shaped by an interconnected set of biological, behavioral, environmental, psychological, and socioeconomic determinants, several of which are potentially modifiable [14]. Among these factors, diet has received increasing attention because dietary intake may influence sleep-regulatory processes while sleep disruption may, in turn, alter food preferences and eating behavior [15]. Previous comprehensive evidence in the literature reported that healthy dietary patterns characterized by greater consumption of plant-based foods were generally associated with better sleep quality [16], whereas diets characterized by processed foods and free sugars were associated with less favorable sleep outcomes [17]. Potential pathways linking higher-quality diets to better sleep include greater intake of fiber, complex carbohydrates, unsaturated fats, and micronutrients involved in sleep regulation; lower intake of added sugars and saturated fat; and favorable effects on postprandial metabolism, oxidative stress, systemic inflammation, and neuroendocrine signaling [18]. These pathways may also intersect with adiposity, insulin resistance, and chronic low-grade inflammation, providing a biological rationale for examining diet–sleep associations according to weight status [19,20].

Against this background, the EAT–Lancet Commission proposed the Planetary Health Diet (PHD) as a reference dietary pattern intended to jointly support human health and environmentally sustainable food systems [21]. The pattern is predominantly plant-based and emphasizes vegetables, fruits, whole grains, legumes, nuts, and unsaturated oils; it permits moderate amounts of fish, poultry, eggs, and dairy products while recommending substantial limitations on red and processed meat, added sugars, and saturated fats [22]. Previous studies assessing the nutritional features of the PHD have shown overall good quality with some potential limitations in animal-derived nutrients, including certain group B vitamins, vitamin D, calcium, and iron [23,24,25,26]. The PHD therefore shares several nutritional characteristics with Mediterranean, DASH, and other high-quality dietary patterns, while explicitly incorporating environmental sustainability into its conceptual framework [27]. The Mediterranean region represents a particularly informative setting in which to examine the PHD. Whereas the Mediterranean diet reflects a culturally and regionally rooted dietary tradition, the PHD was developed as a global reference pattern that explicitly integrates nutritional health with environmental sustainability [28]. Examining PHD adherence in a Mediterranean population may therefore help clarify whether this sustainability-oriented dietary framework is associated with health outcomes even in a setting where a broadly similar plant-forward dietary tradition is already well established.

Epidemiologic evidence concerning the health implications of the PHD has expanded rapidly, providing an association with lower risks of type 2 diabetes, cardiovascular outcomes, cancer mortality, and all-cause mortality, although heterogeneity was substantial for several outcomes and the evidence remained observational [29]. Specifically on weight-related outcomes, comprehensive evidence suggests that greater adherence to sustainable dietary patterns is associated with lower risks of overweight and obesity, supporting a potential connection between planetary-health eating patterns and body-weight regulation [30]. Emerging evidence also extends to mental and neurological health, with studies showing an association between greater EAT–Lancet diet adherence and lower risks of depression and anxiety [31]. Despite this expanding evidence, sleep remains comparatively understudied within the PHD framework, although it represents a multidimensional health behavior potentially linked to dietary exposures [1,15]. Overall, these findings suggest that the PHD may capture dietary characteristics relevant to metabolic, inflammatory, and neurobehavioral processes that are also implicated in sleep regulation, while providing no basis by themselves for inferring a causal effect on sleep. Unlike the Mediterranean diet, these features are incorporated within a dietary framework explicitly developed to jointly address human health and environmental sustainability. In this context, the present study aimed to examine the association between adherence to the PHD and sleep quality among Italian adults living in the Mediterranean basin, using the Pittsburgh Sleep Quality Index (PSQI) to assess both overall sleep quality and individual sleep domains. The present analysis extends the work reported in a previous study in the same cohort in relation to Mediterranean-diet adherence by evaluating the PHD, a distinct dietary framework that incorporates both nutritional and environmental sustainability targets and is operationalized through a different adherence index. In addition, the study explored whether the association between PHD adherence and sleep quality differed according to weight status, thereby providing a more comprehensive assessment of the potential relationship between a sustainable dietary pattern and multidimensional sleep health.

## 2. Materials and Methods

### 2.1. Study Design and Population

This cross-sectional analysis used data from the Mediterranean Healthy Eating, Ageing, and Lifestyles (MEAL) study, a population-based investigation of dietary and lifestyle characteristics among adults living in Sicily, southern Italy [32]. During 2014–2015, men and women aged ≥18 y were randomly selected from the patient lists of general practitioners in the urban area of Catania. The sampling strategy was stratified by municipality area, sex, and 10 y age group. Pregnant women were not eligible. The theoretical sample size was set at 1500 individuals to provide a specific relative precision of 5% (Type I error, 0.05; Type II error, 0.10), taking into account an anticipated 70% participation rate. Of 2405 individuals invited to participate, 2044 completed the study procedures, corresponding to a response rate of 85%. Participants provided written informed consent. The study was conducted in accordance with the Declaration of Helsinki and was approved by the ethics committee of the relevant health authority.

### 2.2. Sociodemographic, Lifestyle, and Anthropometric Information

Face-to-face computer-assisted personal interviews were conducted by trained personnel to collect information on age, sex, educational attainment, occupational status, smoking, alcohol consumption, and physical activity through interviewer-administered questionnaires. Educational attainment was classified as low (primary or secondary school), medium (high school), or high (university). Occupational status was categorized as unemployed, low (unskilled occupations), medium (partially skilled occupations), or high (skilled occupations). Smoking status was classified as never, former, or current smoking. Alcohol consumption was categorized as none, moderate (0.1–12 g/d), or regular (>12 g/d). Physical activity was assessed with the International Physical Activity Questionnaire and categorized as low, moderate, or high [33]. Anthropometric measurements were obtained using standardized procedures. Body mass index (BMI) was calculated as weight in kilograms divided by height in meters squared and categorized as underweight or normal weight (<25 kg/m^2^), overweight (25.0–29.9 kg/m^2^), or obesity (≥30 kg/m^2^).

### 2.3. Dietary Assessment

Habitual dietary intake was assessed using a 110-item semiquantitative food-frequency questionnaire (FFQ) developed and validated for adults living in Sicily [34,35]. The questionnaire assessed average consumption during the preceding 6 months and provided 9 frequency-response categories, ranging from “never” to “4–5 times per day.” Consumption of seasonal foods was reported for the period during which each food was available and was subsequently weighted to estimate average annual intake. Reported frequencies and standard portion sizes were converted into daily food intakes. Energy and nutrient intakes of individual foods and mixed dishes (if available, otherwise disaggregated into individual ingredients) were estimated using the Italian food-composition tables of the Research Center for Foods and Nutrition (CREA). Dietary records with total energy intakes <1000 or >6000 kcal/d were individually reviewed for missing foods or implausible responses (with investigators assessing dietary plausibility unaware of participants’ sleep-quality status). After the exclusion of 107 records considered unreliable, 1936 participants were included in the analytical sample (Supplementary Figure S1).

### 2.4. Planetary Health Diet Index

Adherence to the PHD was evaluated using the PHDI developed by Cacau et al. [23,36]. The index operationalizes the EAT–Lancet reference diet by expressing the intake of its food components relative to total energy intake, thereby permitting comparisons among individuals with different energy requirements. Food items recorded with the FFQ were assigned to the corresponding PHDI components, and the contribution of each component to total energy intake was calculated. The PHDI comprises 16 components organized into 4 scoring categories: Five adequacy components: nuts and peanuts, legumes, fruit, vegetables, and whole cereals; Five optimum components: eggs, fish and seafood, tubers and potatoes, dairy products, and vegetable oils; Two ratio components: dark-green vegetables relative to total vegetables and red-and-orange vegetables relative to total vegetables; Four moderation components: red meat, chicken and substitutes, animal fats, and added sugars. FFQ items assigned to each of the 16 PHDI components are reported in Supplementary Table S1. For adequacy components, intake scores increased proportionally from 0 points in the absence of consumption to 10 points at or above the recommended caloric-density threshold. For optimum components, scores increased from 0 to 10 points up to the reference intake and then decreased proportionally toward 0 when intake exceeded the upper recommended limit. The 2 vegetable-ratio components contributed 0–5 points each, whereas moderation-component scores decreased proportionally from 10 points at no intake to 0 points at or above the respective upper threshold. Intermediate intakes were assigned proportional scores rather than binary values. Component scores were summed to obtain a total ranging from 0 to 150 points, with higher scores indicating closer adherence to the Planetary Health Diet. For the present analysis, participants were categorized into sex-combined, sample-specific quartiles of the total PHDI.

### 2.5. Assessment of Sleep Quality

Sleep quality was assessed using the Pittsburgh Sleep Quality Index (PSQI), a validated 19-item self-report instrument [37]. The items generate 7 component scores: subjective sleep quality, sleep latency, sleep duration, habitual sleep efficiency, sleep disturbances, use of sleep medication, and daytime dysfunction. Each component is scored from 0 to 3, with higher values indicating poorer sleep characteristics. The component scores are summed to obtain a global PSQI score ranging from 0 to 21, with higher scores representing poorer overall sleep quality. Consistent with the previously published analysis, participants with a global PSQI score <5 were classified as having adequate sleep quality, whereas those with a score ≥5 were classified as having inadequate sleep quality.

### 2.6. Statistical Analysis

Mean and standard deviations (SDs) and median and ranges were used to describe central tendency and dispersion. Participant characteristics and sleep features were summarized using frequencies and percentages for categorical variables, with differences across PHDI quartiles examined using chi-square tests. Multivariable logistic regression was used to estimate odds ratios (ORs) and 95% confidence intervals (CIs) for the association between PHDI adherence and adequate sleep quality and its domains. PHDI quartiles were entered as indicator variables, with the lowest quartile as the reference category. The adjusted model included age, sex, BMI, physical activity level, educational attainment, occupational status, smoking status, and total energy intake. To evaluate whether the association differed according to weight status, analyses were repeated within normal-weight and overweight/obesity strata. All tests were 2-sided, and *p* < 0.05 was considered statistically significant. Analyses were conducted using SPSS 29 (SPSS Inc., Chicago, IL, USA).

## 3. Results

The sample reported a mean PHDI score of 66.5 (±12.8 SD). The distribution of background characteristics by level of adherence to the PHDI is presented in Table 1. Participant characteristics differed across PHDI quartiles according to age, smoking status, BMI, occupational status, and physical activity (*p* ≤ 0.008 for all), with participants with higher PHDI adherence generally being older, less likely to be current smokers, and more physically active. Sex and educational attainment did not differ across quartiles.

Sleep quality and individual domains by level of adherence to the PHDI are presented in Table 2. The prevalence of adequate sleep quality increased across PHDI quartiles (*p* = 0.002). Higher PHDI adherence was also associated with better self-rated sleep quality (*p* = 0.037), whereas no significant differences were observed for the other sleep domains.

The association between adherence to the PHD and sleep quality and its individual features is shown in Table 3. In fully adjusted models, participants in Q2 and Q4 had greater odds of adequate sleep quality than those in Q1 [OR: 1.47 (95% CI: 1.06, 2.03) and OR: 1.65 (95% CI: 1.20, 2.28), respectively; Table 3]. Compared with Q1, participants in Q2, Q3, and Q4 had higher odds of better self-rated sleep quality. Selected quartile-specific associations were also observed for favorable sleep-disturbance profiles in Q2 and Q4 and for day dysfunction and sleep latency in Q2. No significant associations were found for sleep duration, sleep efficiency, or use of sleep medication.

An analysis stratified by weight status is provided in Table 4. No consistent association between PHDI adherence and adequate sleep quality was observed among participants with normal weight (Table 4). Conversely, among participants with overweight or obesity, all higher PHDI quartiles were associated with greater odds of adequate sleep quality, with the largest estimate observed in Q4 [OR: 2.40 (95% CI: 1.46, 3.95)]. In this subgroup, associations with day dysfunction were observed across Q2, Q3, and Q4, whereas selected quartile-specific associations were observed for sleep efficiency and self-rated sleep quality in Q4 and for sleep-disturbance profiles and sleep latency in Q2. No associations were observed for sleep duration or use of sleep medication.

## 4. Discussion

In this cross-sectional study on Italian adults, greater adherence to the PHD was associated with a higher likelihood of adequate overall sleep quality. About two-thirds of individuals in the highest quartile of adherence to the PHD reported adequate sleep quality, resulting in a 65% higher likelihood of adequate sleep quality. Among the individual PSQI domains, subjective sleep quality showed the most internally consistent association with PHDI adherence. In contrast, associations with sleep latency, sleep disturbance, and daytime dysfunction were observed only in selected quartiles and lacked a clear graded pattern; these findings should therefore be interpreted as exploratory. No association was found with sleep duration or the use of sleep medications. Although the overall pattern across PHDI quartiles was not strictly monotonic, participants in the highest quartile had a greater likelihood of adequate sleep quality than those in the lowest quartile. In analyses stratified by weight status, the associations were more pronounced among participants with overweight or obesity, in whom higher PHDI quartiles were associated with adequate overall sleep quality and several individual sleep dimensions. Conversely, no clear association with overall sleep quality was observed among participants with normal weight.

Evidence specifically addressing the PHD and sleep remains limited but is emerging. To date, only a few studies have directly examined this association. In a large cross-sectional study of older Chinese adults (aged ≥ 65 years) from the Chinese longitudinal healthy longevity survey, participants in the highest PHDI tertile had approximately 35% higher odds of reporting good sleep quality than those in the lowest tertile [38]. The magnitude of this association was somewhat lower than that observed in the present study, where participants in the highest PHDI quartile had approximately 65% higher odds of adequate overall sleep quality and 67% higher odds of better self-rated sleep quality compared with those in the lowest quartile, although direct comparisons should be made cautiously. The Chinese study included only older adults and used a simplified PHDI based on 10 food groups scored according to consumption frequency and categorized into tertiles, whereas the present study included adults across a broader age range and applied a proportional 16-component PHDI categorized into quartiles. Sleep assessment also differed, as the Chinese study relied on a single self-reported item for sleep quality, while the present study used the multidimensional PSQI. The association with adequate sleep duration reported in the Chinese study was not reproduced in the present analysis. Sleep duration may capture a different dimension of sleep health from subjective sleep quality, as sleep is a multidimensional construct extending beyond duration alone [39]. Moreover, the use of self-reported and categorized sleep duration may have reduced sensitivity to detect associations with PHD adherence. Differences in age, the construction of the dietary index, the use of a simplified FFQ, and the definition of sleep outcomes may explain this discrepancy. More recently, a prospective UK Biobank analysis of 165,866 adults with a mean baseline age of 55.8 years reported lower hazards of clinically recorded sleep disorders among participants with higher PHD adherence, with HRs of 0.827, 0.796, and 0.838 across the three different PHD scoring systems, respectively [40]. This study differed substantially from the present analysis, as dietary intake was assessed using 24 h dietary recalls and sleep outcomes were based on ICD-10 diagnoses rather than perceived sleep quality. Moreover, PHD adherence was quantified using three distinct scoring approaches, which, together with differences in exposure categorization, limits direct comparison of adherence ranges and effect estimates with the present study. Together, these studies provide convergent but still preliminary evidence linking the PHD with sleep health. On a broad level, evidence on other dietary patterns sharing similar features of the PHD results is rather consistent with the present findings. The Mediterranean diet is the dietary pattern most extensively investigated in relation to sleep, owing to its emphasis on fruits, vegetables, legumes, nuts, whole grains, fish, and olive oil and its comparatively low content of red and processed meat, refined foods, and added sugars [41,42]. A recent summary of evidence found that better sleep quality, sufficient sleep duration, and an earlier chronotype were associated with greater Mediterranean-diet adherence [43,44,45]. Associations with sleep outcomes have also been reported for other healthy dietary patterns, such as the Dietary Approaches to Stop Hypertension (DASH) pattern, the Healthy Eating Index (HEI), and plant-rich diets, although these bodies of evidence are less developed than that for the Mediterranean diet [46]. Similarly, higher HEI scores were associated with lower odds of abnormal sleep duration [47]. Observational evidence has also linked healthful plant-based dietary patterns with a lower incidence of insomnia, although potentially different associations have been reported in men and women [48]. Conversely, dietary features emphasizing pro-inflammatory processes have been comprehensively associated with lower sleep quality [49]. In interpreting these findings, the conceptual overlap between the PHD and the Mediterranean diet should also be considered, as both capture several features of a high-quality, plant-forward dietary pattern [50]. Nonetheless, higher adherence to the PHD is more restrictive toward animal-based products compared to the Mediterranean diet, which could impact the intake of amino acids, such as tryptophan and methionine, which are known to promote sleep quality [51,52]. However, such a hypothesis was not confirmed, as a positive association with sleep quality was equally observed for individuals more adherent to the PHD, suggesting that other elements, including plant-derived minerals, vitamins, and phytochemicals, may also promote brain health [53,54]. Thus, the present results appear to form part of a more general relationship between plant-forward, nutrient-dense dietary patterns and sleep health rather than an association unique to a single dietary score. Accordingly, the observed association may reflect, at least in part, features shared with other high-quality, plant-rich dietary patterns, while the potential contribution of characteristics specific to the PHD cannot be established from the present study.

The findings concerning weight status, although only exploratory given that no formal interaction test was performed, warrant particular consideration. A greater burden of sleep disturbances is generally reported in the scientific literature in individuals with overweight and obesity, possibly due to mechanical, metabolic, inflammatory, and psychological pathways, including an increased susceptibility to obstructive sleep apnea [55]. Individuals with excess adiposity may therefore have greater metabolic or inflammatory scope to benefit from a high-quality dietary pattern. Consistent with this possibility, the aforementioned Chinese survey reported a significant multiplicative interaction between PHDI and BMI for sleep quality, with PHDI being associated with both higher sleep duration and quality in individuals with higher BMI [38]. Moreover, the UK Biobank study reported that BMI partially mediated the association between greater PHD adherence and lower incidence of diagnosed sleep disorders [40]. From the broader perspective of studies conducted on healthy dietary patterns, another study conducted on Chinese young adults reported that the association between higher adherence to a fruit-, vegetable-, and dairy-rich dietary pattern and sleep quality was numerically strongest among participants with BMI ≥25 kg/m^2^, although BMI did not significantly modify the association [56]. Similarly, another study in US adults showed that higher diet quality (higher alternate-healthy eating index scores) was associated with lower obstructive sleep apnea risk, with the associations stronger in those with BMI ≥25 kg/m^2^ than for their counterparts [57]. In contrast, although an earlier analysis conducted in the same sample included in the present study showed that greater Mediterranean diet adherence was associated with better sleep quality, the weight-stratified findings differed from the present ones, with associations reported among participants with normal weight or overweight but not among those with obesity [58]. Hence, the consistency of the results, albeit not independent, suggests that shared features of the Mediterranean and planetary health diets may be relevant to sleep, but with different effect modification by body weight status. Another study conducted on Iranian adults reported similar findings, with stratified analysis showing stronger associations between a plant-based dietary pattern and adequate sleep duration and quality in normal-weight rather than participants with obesity [59]. Finally, a study conducted on middle-aged adults from the CoLaus PsyCoLaus, a population-based study in Lausanne, Switzerland, reported no actual changes in the association between adherence to the Mediterranean diet and sleep quality in the analyses stratified for BMI categories [60]. Hence, the general findings on the role of weight status are rather conflicting. From a biological perspective, individuals with excess adiposity may have a greater burden of systemic inflammation, impaired glucose regulation and sleep-disordered breathing [61], potentially making control over obesity-related inflammation and the metabolically favorable characteristics of a plant-rich diet more relevant to their sleep health [62]. Conversely, in individuals with more severe obesity, mechanical obstruction of the upper airways, comorbidities, and other obesity-related determinants of sleep may predominate over the comparatively modest contribution of diet, potentially explaining the null associations reported in some obese subgroups [63]. Importantly, undiagnosed obstructive sleep apnea may represent a source of residual confounding in the weight-stratified analyses since it is primarily related to higher BMI but not dietary features. Thus, the associations observed among participants with overweight or obesity in the present study should be considered suggestive of greater metabolic susceptibility rather than evidence of a universally stronger dietary effect in this group. However, combining overweight and obesity into a single category may have masked potential differences in the observed associations between these two BMI groups. These findings extend the limited literature on the interplay among sustainable dietary patterns, adiposity and sleep, but require confirmation in prospective studies incorporating objective measures of adiposity and sleep and adequately powered formal interaction analyses.

Several interrelated mechanisms could plausibly link PHD adherence with sleep. First, the PHD promotes vegetables, fruits, whole grains, legumes, and nuts, while limiting red and processed meat, added sugars, and other sources of saturated fat [36]. This combination results in greater intakes of dietary fiber, (poly)phenols, and unsaturated fatty acids and a lower inflammatory potential and may reduce selected inflammatory biomarkers, although heterogeneity remains considerable [64]. Because low-grade systemic inflammation and oxidative stress are involved in sleep regulation and are commonly elevated in obesity, attenuation of these processes represents a biologically plausible [65,66], but untested, explanation for the more pronounced associations observed among participants with overweight or obesity [67]. Second, the foods prioritized in the PHD provide substrates and cofactors involved in neurotransmitter and circadian regulation. Tryptophan, B-group vitamins, and unsaturated fatty acids may influence pathways involved in neuronal signaling and sleep regulation [68,69]. Nevertheless, the effects of individual nutrients depend on the overall food matrix, meal composition, timing, and metabolic context, and direct evidence that these nutrients mediate associations between whole dietary patterns and sleep remains limited. The lower content of added sugars and refined foods in a high-PHDI diet may also reduce glycemic fluctuations and other metabolic disturbances that could contribute to nocturnal awakenings or impaired sleep continuity [70]. Third, a fiber-rich diet may influence sleep through the gut–brain axis. Fermentable carbohydrates support microbial production of short-chain fatty acids, whereas diet-related changes in microbial tryptophan metabolism, bile acids, immune signaling, and intestinal barrier function may affect neuroendocrine and circadian pathways [71]. However, a recent overview of human studies found heterogeneous relationships between sleep, circadian health, the gut microbiome, and related inflammatory mechanisms, with relatively few studies measuring microbial metabolites or testing causal pathways [72,73]. The microbiome should therefore be regarded as a promising mechanistic hypothesis rather than an established mediator. Additionally, psychological health may constitute an additional pathway. Greater adherence to EAT–Lancet dietary patterns has been associated with lower risks of depression and anxiety, although the certainty of this evidence remains low and is largely based on observational studies [31]. Because depressive and anxiety symptoms are closely related to perceived sleep quality and daily dysfunction, mental health could partly mediate, or confound, the observed association. Depression and anxiety were not specifically accounted for in the present analysis, and this possibility should be examined in future studies. Finally, the relationship between diet and sleep is likely bidirectional. Experimental evidence indicates that sleep restriction can result in higher energy intakes and may alter macronutrient intakes [74], while the selection of energy-dense, highly palatable foods, like ultra-processed foods, reduces the capacity to maintain healthy lifestyle behaviors and is related to adverse sleep outcomes [75]. Consequently, participants with better sleep may have been more likely to follow a high-quality diet, rather than, or in addition to, a high-quality diet contributing to better sleep.

The present study has several strengths. Participants were recruited from the general population using a random sampling strategy; the sample was relatively large, and dietary intake was assessed using a validated food-frequency questionnaire. Adherence was evaluated using a proportional PHDI that captures several complementary dimensions of the EAT–Lancet reference diet, while sleep was assessed using the validated PSQI, allowing overall sleep quality and its individual components to be examined. The analyses also accounted for a broad range of sociodemographic, lifestyle, anthropometric, clinical, and dietary covariates, and the stratified analyses permitted the association to be explored across weight-status groups.

However, some limitations should also be acknowledged. The cross-sectional design precludes determination of temporality and causality, which is particularly important given the bidirectional relationship between diet and sleep. Both dietary intake and sleep were self-reported and are therefore susceptible to recall error, social-desirability bias, and exposure or outcome misclassification. Missing data and potential complete-case selection bias in fully adjusted models excluding participants with incomplete covariate information may be another methodological limitation to be taken into account. The absence of objective sleep measurements, such as actigraphy or polysomnography, prevented evaluation of sleep architecture and the identification of undiagnosed obstructive sleep apnea. Residual confounding by depression and anxiety cannot be excluded and may be particularly relevant for subjective sleep quality and daytime dysfunction, given their close relationship with psychological symptoms. Caffeine and alcohol intake may also represent a particularly important source of residual confounding because of their potential influence on sleep. Other potential residual confounders include stress, chronotype, shift work, sleep hygiene, screen time, meal timing, sleep-related medications, visceral adiposity, and additional unmeasured factors. However, given the relatively small sample, some variables have been ruled out from the final analysis to avoid overadjustment. Higher PHD adherence may also be a marker of a broader health-conscious lifestyle that was not fully captured by the available covariates. In addition, the component-level and stratified analyses involved multiple statistical comparisons, increasing the possibility of chance findings. Some associations were confined to selected intermediate quartiles and did not show a strictly graded pattern. The apparent difference between weight-status groups was not supported by a reported interaction test and should therefore be interpreted cautiously. Moreover, stratification reduced the sample size, potentially weakening the power of the analyses. The PHDI quantifies dietary adherence but does not directly measure the environmental effects of each participant’s diet; accordingly, environmental benefits cannot be inferred from the present health analysis. Finally, participants were recruited from an urban area of Sicily, which may limit generalizability to populations with different dietary traditions, food systems, socioeconomic conditions, or sleep patterns.

## 5. Conclusions

In the present study, greater adherence to the Planetary Health Diet was associated with better overall and self-rated sleep quality in Italian adults, with associations appearing more evident among participants with overweight or obesity. These findings are consistent with the limited emerging evidence directly linking PHD adherence with sleep and with the broader literature on Mediterranean, plant-forward, and anti-inflammatory dietary patterns. Nevertheless, the cross-sectional design, reliance on self-reported sleep measures, and exploratory nature of the weight-stratified analyses preclude causal conclusions. From a clinical perspective, the present findings suggest that adherence to a high-quality dietary pattern such as the PHD may represent a relevant lifestyle factor to consider in the broader assessment of sleep health. Nevertheless, prospective and interventional evidence is required before PHD adherence can be translated into specific dietary recommendations for sleep improvement. Randomized dietary interventions incorporating repeated dietary assessments, actigraphy or polysomnography, and metabolic and inflammatory biomarkers are needed. If confirmed, the findings would support the hypothesis that dietary strategies intended to support both human and planetary health may provide potential co-benefit for sleep health.

## Figures and Tables

**Table 1:** Background characteristics of the study participants by quartiles of Planetary Health Diet Index score.

	PHDI Score				
	Q1	Q2	Q3	Q4	*p*-Value
**PHDI** **score, median (range)**	53.3 (4.3–59.0)	62.9 (59.0–66.1)	70.0 (66.1–74.6)	81.3 (74.7–112.7)	-
**Sex, n (%)**					0.437
Men	187 (40.0)	216 (44.1)	194 (39.5)	207 (42.4)	
Women	280 (60.0)	274 (55.9)	297 (60.5)	281 (57.6)	
**Age, n (%)**					<0.001
<30 y	150 (32.1)	130 (26.5)	105 (21.4)	121 (24.8)	
30–49 y	142 (30.4)	144 (29.4)	147 (29.9)	114 (23.4)	
50–69 y	100 (21.4)	122 (24.9)	132 (26.9)	120 (24.6)	
≥70 y	75 (16.1)	94 (19.2)	107 (21.8)	133 (27.3)	
**Smoking status, n (%)**					<0.001
Never	256 (54.8)	286 (58.4)	340 (69.2)	313 (64.1)	
Former	132 (28.3)	119 (24.3)	91 (18.5)	123 (25.2)	
Current	79 (16.9)	85 (17.3)	60 (12.2)	52 (10.7)	
**BMI, n (%)**					0.008
Normal	207 (49.2)	182 (40.8)	215 (46.6)	247 (52.6)	
Overweight	139 (33.0)	176 (39.5)	155 (33.5)	160 (34.0)	
Obese	75 (17.8)	88 (19.7)	91 (19.7)	63 (13.4)	
**Educational level, n (%)**					0.214
Low	176 (37.7)	173 (35.3)	180 (36.7)	168 (34.4)	
Medium	158 (33.8)	173 (35.3)	196 (39.9)	193 (39.5)	
High	133 (28.5)	144 (29.4)	115 (23.4)	127 (26.0)	
**Occupational level, n (%)**					<0.001
Unemployed	111 (26.8)	105 (26.5)	121 (29.2)	124 (28.6)	
Low	47 (11.4)	64 (16.2)	78 (18.8)	77 (17.7)	
Medium	147 (35.5)	93 (23.5)	104 (25.1)	96 (22.1)	
High	109 (26.3)	134 (33.8)	111 (26.8)	137 (31.6)	
**Physical activity level, n (%)**					<0.001
Low	73 (18.3)	89 (20.3)	87 (19.5)	80 (18.0)	
Medium	233 (58.4)	209 (47.6)	228 (51.1)	186 (41.9)	
High	93 (23.3)	141 (32.1)	131 (29.4)	178 (40.1)	

**Table 2:** Distribution of overall sleep quality and individual domains of sleep characteristics by quartiles of Planetary Health Diet Index score.

	PHDI Score				
	Q1	Q2	Q3	Q4	*p*-Value
**Sleep quality, n (%)**					0.002
Adequate	284 (60.8)	340 (69.4)	342 (69.7)	348 (71.3)	
Inadequate	183 (39.2)	150 (30.6)	149 (30.3)	140 (28.7)	
**Sleep efficiency, n (%)**					0.180
High	154 (33.0)	137 (28.0)	144 (29.3)	131 (26.8)	
Low	313 (67.0)	353 (72.0)	347 (70.7)	357 (73.2)	
**Sleep duration, n (%)**					0.581
Adequate	193 (41.3)	200 (40.8)	197 (40.1)	182 (37.3)	
Inadequate	274 (58.7)	290 (59.2)	294 (59.9)	306 (62.7)	
**Sleep disturbance, n (%)**					0.214
Low	424 (90.8)	426 (86.9)	441 (89.8)	429 (87.9)	
High	43 (9.2)	64 (13.1)	50 (10.2)	59 (12.1)	
**Self-rated sleep quality, n (%)**					0.037
Low	387 (82.9)	382 (78.0)	377 (76.8)	369 (75.6)	
High	80 (17.1)	108 (22.0)	114 (23.2)	119 (24.2)	
**Need medication to sleep, n (%)**					0.567
Yes	53 (11.3)	49 (10.0)	48 (9.8)	42 (8.6)	
No	414 (88.7)	441 (90.0)	443 (90.2)	446 (91.4)	
**Day dysfunction, n (%)**					0.080
Low	225 (48.2)	196 (40.0)	222 (45.2)	213 (43.6)	
High	242 (51.8)	294 (60.0)	269 (54.8)	275 (56.4)	
**Sleep latency, n (%)**					0.061
Short	279 (59.7)	256 (52.2)	283 (57.6)	260 (53.3)	
Long	188 (40.3)	234 (47.8)	208 (42.4)	228 (46.7)	

**Table 3:** Association between overall and individual domains of sleep quality and adherence to the Planetary Health Diet Index score. The results represent a fully adjusted model including age, sex, BMI, physical activity level, educational attainment, occupational status, smoking status, and total energy intake.

	PHDI, OR (95% CI)			
	Q1	Q2	Q3	Q4
Sleep quality (adequate)	1	1.47 (1.06–2.03) *	1.35 (0.98–1.85)	1.65 (1.20–2.28) *
Sleep efficiency (high)	1	1.34 (0.96–1.86)	1.25 (0.90–1.73)	1.32 (0.95–1.83)
Sleep duration (adequate)	1	0.95 (0.70–1.29)	1.01 (0.74–1.37)	1.18 (0.64–1.60)
Sleep disturbance (low)	1	2.11 (1.29–3.46) *	1.51 (0.90–2.53)	1.68 (1.01–2.79) *
Self-rated sleep quality (high)	1	1.64 (1.11–2.41) *	1.63 (1.11–2.40) *	1.67 (1.14–2.46) *
Need medication to sleep (no)	1	1.12 (0.66–1.89)	0.82 (0.50–1.34)	1.20 (0.71–2.04)
Day dysfunction (low)	1	1.38 (1.02–1.87) *	1.15 (0.85–1.55)	1.23 (0.91–1.67)
Sleep latency (short)	1	1.40 (1.03–1.91) *	1.08 (0.80–1.47)	1.33 (0.98–1.81)

* denotes *p* < 0.05.

**Table 4:** Association between overall and individual domains of sleep quality and adherence to the Planetary Health Diet Index score by weight status. The results represent a fully adjusted model including age, sex, physical activity level, educational attainment, occupational status, smoking status, and total energy intake.

	PHDI, OR (95% CI)			
	Q1	Q2	Q3	Q4
**Normal weight**				
Adequate sleep quality	1	1.22 (0.72–2.05)	0.97 (0.59–1.58)	1.14 (0.71–1.84)
Sleep efficiency	1	1.66 (0.96–2.88)	1.11 (0.67–1.82)	1.06 (0.66–1.71)
Sleep duration	1	0.92 (0.56–1.49)	0.95 (0.59–1.53)	1.11 (0.70–1.75)
Sleep disturbance	1	1.17 (0.72–1.89)	0.85 (0.53–1.36)	1.23 (0.79–1.92)
Self-rated sleep quality	1	1.80 (1.00–3.23) *	1.65 (0.93–2.93)	1.57 (0.90–2.74)
Need medication to sleep	1	0.99 (0.44–2.26)	0.66 (0.31–1.41)	1.12 (0.51–2.47)
Day dysfunction	1	1.23 (0.75–2.01)	0.81 (0.51–1.28)	0.97 (0.62–1.51)
Sleep latency	1	1.17 (0.72–1.89)	0.85 (0.53–1.36)	1.23 (0.79–1.92)
**Overweight/obese**				
Adequate sleep quality	1	1.60 (1.00–2.56) *	1.61 (1.00–2.57) *	2.40 (1.46–3.95) *
Sleep efficiency	1	1.18 (0.73–1.90)	1.25 (0.77–2.02)	1.69 (1.02–2.79) *
Sleep duration	1	0.97 (0.62–1.53)	1.042 (0.66–1.63)	1.18 (0.74–1.88)
Sleep disturbance	1	2.04 (1.01–4.12) *	1.03 (0.48–2.23)	1.53 (0.72–3.24)
Self-rated sleep quality	1	1.68 (0.95–2.98)	1.47 (0.82–2.64)	1.79 (1.01–3.20) *
Need medication to sleep	1	0.94 (0.43–2.05)	0.94 (0.44–1.98)	1.23 (0.55–2.74)
Day dysfunction	1	1.86 (1.18–2.93) *	1.62 (1.03–2.57) *	1.63 (1.03–2.57) *
Sleep latency	1	1.72 (1.09–2.70) *	1.19 (0.76–1.88)	1.42 (0.89–2.25)

* denotes *p* < 0.05.

## Data Availability

The original contributions presented in this study are included in the article. Further inquiries can be directed to the corresponding author.

## Supplementary Materials

The following supporting information can be downloaded at https://www.mdpi.com/article/10.3390/nu18213586/s1, Figure S1: Flowchart of participant selection process. Table S1: The Planetary Health Diet Index components and corresponding food frequency questionnaire items included.
