# Supplementary material for: Planetary Health Diet, Obesity and Sleep Quality in Italian Adults
Source: Nutrients. 2026 Oct 29;18(21):3586. doi: 10.3390/nu18213586 (PMC13764929; doi:10.3390/nu18213586)
Supplement: Supplementary file 1 [file nutrients-18-03586-s001.zip › nutrients-4575320-SI.pdf]

Supplementary Figure S1. Flow-chart of participant selection process.

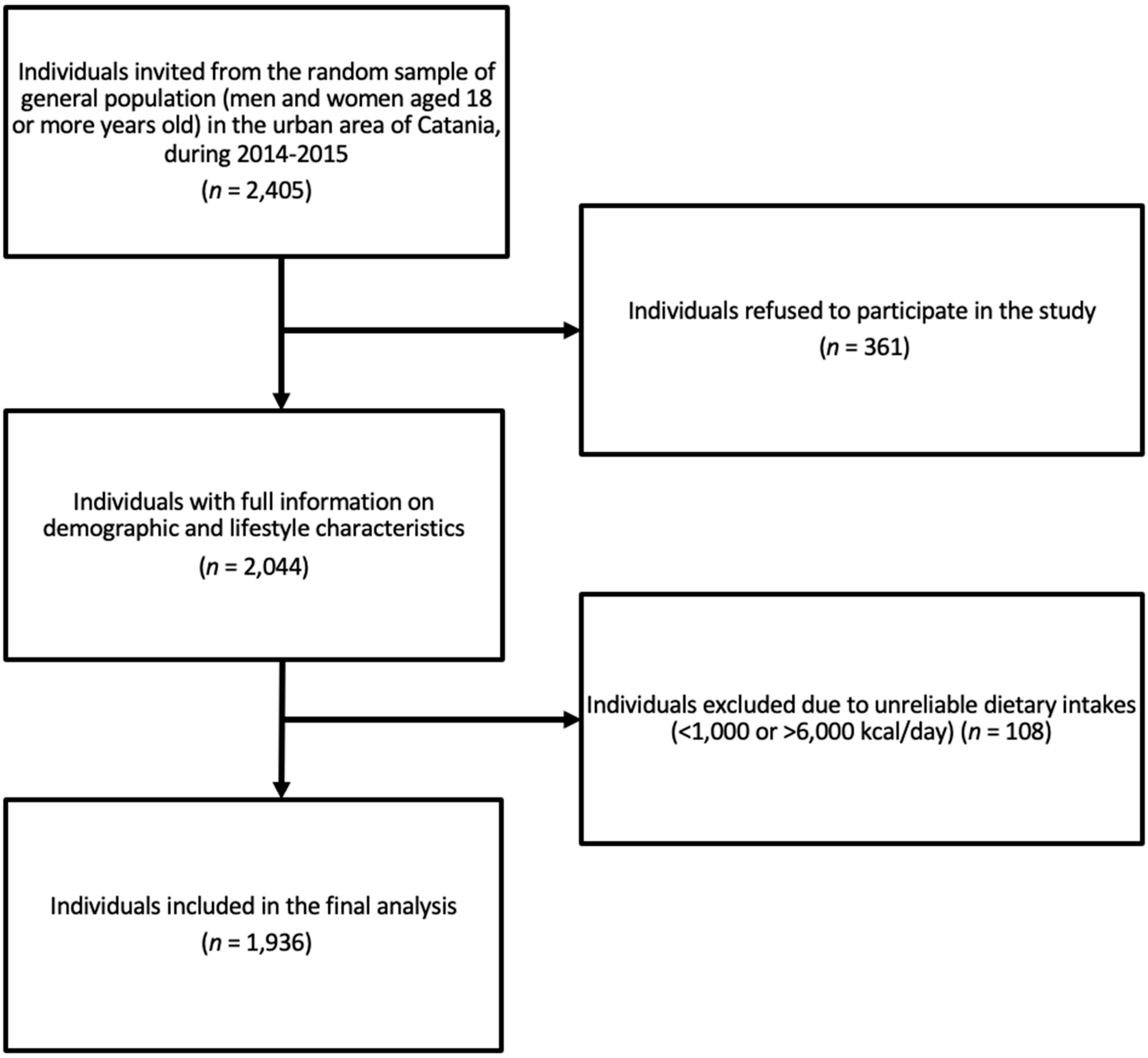

.

Supplementary Table S1. The Planetary Health Diet Index components and corresponding food frequency questionnaire items included.

Components	Corresponding FFQ items
**Adequacy components**	
Fruit	Apple, Pear, Orange, Mandarins, Blood orange, Banana, Grapes, Melon, Watermelon, Peaches, Apricots, Strawberries, Kiwi, Cherries, Blueberries, Currants, Mulberries, Raspberries, Pomegranate, Prickly pears, Plums, Figs, 100% fruit juices or smoothies
Vegetables	Cauliflower, Green beans, Green and white zucchini, Asparagus, Onions, Garlic, Mushrooms, Green salad, Lettuce, Fennel, Artichokes, Bell peppers, Eggplant, Spinach, Broccoli, Cabbage, Brussels sprouts, Chicory, Carrots, Tomatoes
Nuts and peanuts	Peanuts, Pistachios, Walnuts, Almonds, Hazelnuts
Whole cereals	Whole grain pasta, bread, rice
Legumes	Beans, Chickpeas, Peas, Lentils, Fava beans, Soy burgers, Soy Milk, Tofu
**Optimum components**	
Eggs	Eggs
Dairy	Milk, Yogurt, Cheddar, Brie, Caciotta, Parmesan, Ricotta, Low-fat soft cheese, Mozzarella
Tubers and potatoes	Tubers and potatoes, fries
Vegetable oils	Olive oil, seed oil
Fish and seafood	White fish, Cod, Sole, Red mullet, Mackerel, Chub mackerel, Swordfish, Anchovies, Tuna, Salmon, Shellfish, Crustaceans, Shrimp, Mussels
**Ratio components**	
DGV/total vegetables	Spinach, Broccoli, Cabbage, Brussels sprouts, Chicory
ReV/total vegetables	Carrots, Tomatoes
**Moderation components**	
Red meat	Red meat, Pork, Fast food burger
Chicken and substitutes	White meat (chicken, rabbit)
Animal fats	Butter, Cream cheese
Added sugars	Table sugar, Jams and honey, SSBs

DGV, dark green vegetables; FFQ, food frequency questionnaire; ReV, red and orange vegetables; SSBs, sugary-sweetened beverages.
